# Protocol for haplotype-resolved structural variant detection via long-read sequencing using cuteHap

**DOI:** 10.1016/j.xpro.2026.104810

**Published:** 2026-08-28

**Authors:** Shuqi Cao, Chuanmin Wu, Yuejin He, Tao Jiang

**Affiliations:** 1 Faculty of Computing, Harbin Institute of Technology, Harbin, Heilongjiang 150001, China; 2 Zhengzhou Research Institute, Harbin Institute of Technology, Zhengzhou, Henan 450000, China

**Keywords:** Bioinformatics, Genomics, Sequence analysis, Sequencing

## Abstract

Long-read sequencing technologies have revolutionized human genome exploration at an unparalleled resolution, particularly facilitating the analysis of structural variation (SV) at haplotype resolution. Here, we present a protocol for using cuteHap, a robust framework for haplotype-aware SV detection through phased alignment reads generated by diverse long-read sequencing platforms. We describe procedures for single-nucleotide variant (SNV) calling, read phasing, SV calling, and genotyping. We also establish a benchmarking pipeline to evaluate the detected SV callsets.

For complete details on the use and execution of this protocol, please refer to Cao et al.^1^

## Highlights

• Instructions for SNV calling: use long-read based SNV caller, Clair3, to produce SNVs
• Steps for read phasing: execute LongPhase to generate haplotagged alignments
• Guidance on SV calling: execute cuteHap to detect haplotype-resolved SVs
• Optional benchmarking: evaluate against high-confidence ground truth using Truvari

## Abstract (editor highlights)

Publisher’s note: Undertaking any experimental protocol requires adherence to local institutional guidelines for laboratory safety and ethics.

## Graphical abstract

**Figure:**
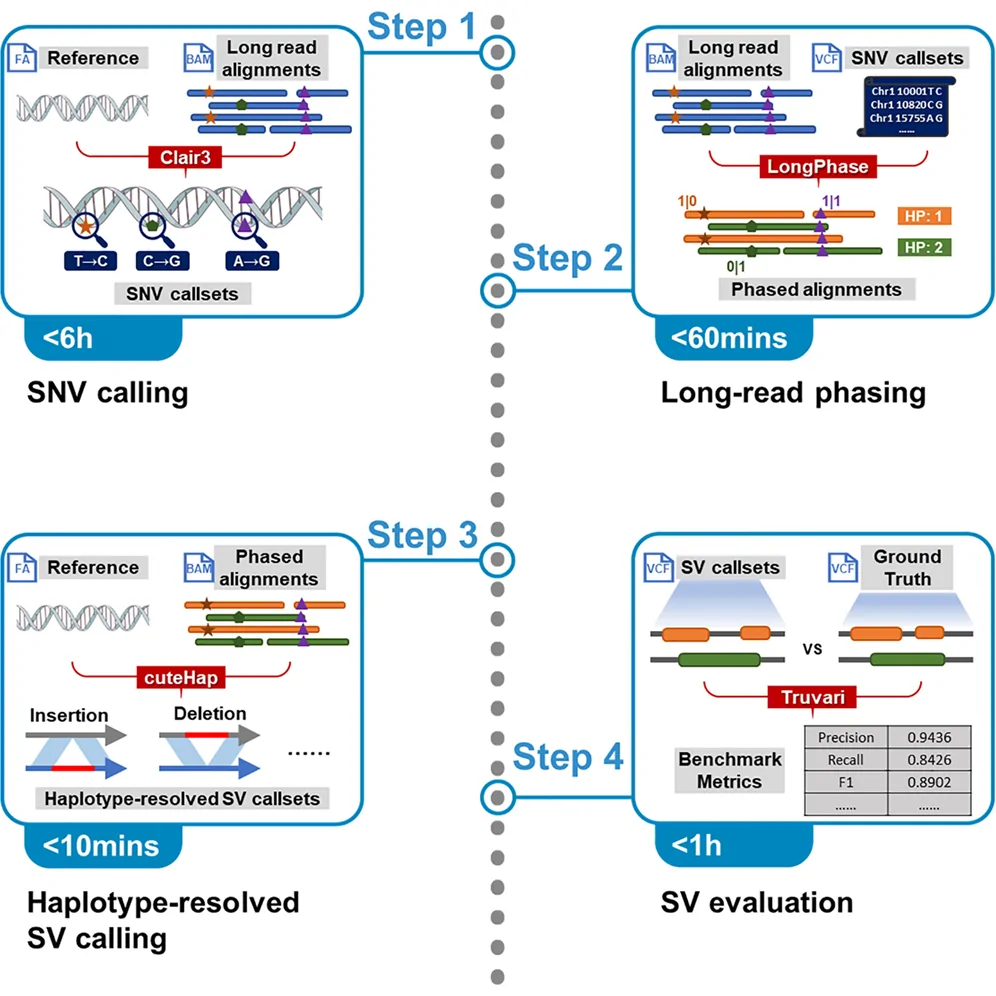

## Before you begin

Structural variations (SVs), defined as genomic alterations of at least 50 bp,^2^ impact cellular processes,^3^ phenotypes,^4^ and diseases.^5^ Long-read sequencing enables comprehensive SV detection as well as higher-resolution haplotype analysis,^6^ which is crucial for elucidating allele-specific effects^7^ and diagnosing recessive disorders.^8^ Long-read-based SV detectors such as cuteSV^9^^,^^10^ and Sniffles2^11^ face bottlenecks in haplotype analysis and accurate recognition in multi-allelic regions. While ensemble methods improve accuracy, their performance depends on the underlying detector. Assembly-based approaches achieve the highest resolution but require substantial computational resources. Currently, an increasing number of variant calling methods are leveraging read phasing information to enhance variant detection and generate haplotype-resolved variant call sets. However, the widespread application of existing phasing-based methods has been hindered by limited cross-platform compatibility, high computational burden, and insufficient utilization of haplotype information. Advancements in phasing-integrated SV calling are essential to overcome these challenges and unlock the full potential of SV analysis for biology and medicine.

Here, we introduce a user-friendly protocol for achieving haplotype-resolved SV calling using sequencing data from any long-read sequencing platform. The protocol below outlines the required steps, which includes single nucleotide variant (SNV) calling, read phasing, and haplotype-resolved SV calling and genotyping. The protocol describes the specific steps using Pacific Biosciences High-Fidelity (PacBio HiFi) sequencing data from the HG002 individual; however, it can also be applied to other individuals with long-read sequencing data.

### Innovation

While long-read sequencing has revolutionized SV detection, existing approaches still face critical bottlenecks in haplotype-aware analysis. Alignment-based SV detectors frequently struggle with accurate detection in multi-allelic regions and reliable haplotype phasing. Ensemble methods, though improving overall accuracy, remain dependent on the performance of individual callers and exhibit similar deficiencies in haplotype assignment. Assembly-based strategies offer high resolution but incur prohibitive computational costs, rendering them impractical for routine application. Recent phasing-integrated tools have emerged to address these issues; however, their broader adoption is hampered by limited cross-platform compatibility, excessive computational demands, and suboptimal utilization of haplotype information.

Herein, we present a protocol through cuteHap, a haplotype-aware SV detection framework that fundamentally reimagines how phased information is leveraged. Its two synergistic innovations, including haplotype-specific self-adaptive clustering and a cluster credibility-prioritized beam search algorithm, collectively enable superior SV discovery and precise phased genotype assignment without sacrificing computational efficiency. Based on this algorithmic breakthrough, our protocol delivers a comprehensive, platform-agnostic workflow for haplotype-resolved SV calling. By detailing an end-to-end pipeline—from SNV calling and read phasing to haplotype-aware SV detection and genotyping—it empowers researchers to apply this advanced framework to any long-read dataset of different technologies. This integration of algorithmic innovation with universal accessibility establishes a new avenue for SV analysis, unlocking the full potential of haplotype-resolved genomics in both research and clinical settings.

### Create working directory

**Timing: 1 min**

1. Create directory structure for generating temporary files and storing results.

> mkdir -p svwork

> mkdir -p svwork/{ref,alns,snvs,phased,svs,benchmarks,models}

### Download the dataset

**Timing: 1–12 h**

2. Download long-read sequencing alignments from PacBio or Oxford Nanopore Technologies (ONT) platforms.

***Note:*** Here, we use the publicly available alignments from PacBio HiFi sequencing data, which were previously aligned to the GRCh38 reference genome. Troubleshooting 1.

> wget -c -P alns https://downloads.pacbcloud.com/public/revio/2022Q4/HG002-rep1/analysis/HG002.m84011_220902_175841_s1.GRCh38.bam

> wget -c -P alns https://downloads.pacbcloud.com/public/revio/2022Q4/HG002-rep1/analysis/HG002.m84011_220902_175841_s1.GRCh38.bam.bai

3. Download the human reference genome and its index.

***Note:*** Here, we use the GRCh38 reference genome.

> wget -c -P ref http://ftp.1000genomes.ebi.ac.uk/vol1/ftp/technical/reference/GRCh38_reference_genome/GRCh38_full_analysis_set_plus_decoy_hla.fa

> wget -c -P ref http://ftp.1000genomes.ebi.ac.uk/vol1/ftp/technical/reference/GRCh38_reference_genome/GRCh38_full_analysis_set_plus_decoy_hla.fa.fai

**CRITICAL:** The reference genome used here must be identical to the one used for the sequencing alignments in the previous step. In this protocol, both the reference genome and the target genome of the sequencing alignments are GRCh38.

4. Download the HG002 benchmark ground truth and the corresponding high-confidence region for subsequent evaluation.

***Note:*** Here, we use the callset released by T2T Q100 based on the GRCh38 reference.

> wget -c -P benchmarks https://ftp-trace.ncbi.nlm.nih.gov/ReferenceSamples/giab/data/AshkenazimTrio/analysis/NIST_HG002_DraftBenchmark_defrabbV0.019-20241113/GRCh38_HG2-T2TQ100-V1.1_stvar.vcf.gz

> wget -c -P benchmarks https://ftp-trace.ncbi.nlm.nih.gov/ReferenceSamples/giab/data/AshkenazimTrio/analysis/NIST_HG002_DraftBenchmark_defrabbV0.019-20241113/GRCh38_HG2-T2TQ100-V1.1_stvar.vcf.gz.tbi

> wget -c -P benchmarks https://ftp-trace.ncbi.nlm.nih.gov/ReferenceSamples/giab/data/AshkenazimTrio/analysis/NIST_HG002_DraftBenchmark_defrabbV0.019-20241113/GRCh38_HG2-T2TQ100-V1.1_stvar.benchmark.bed

5. Download the corresponding pretrained model for Clair3.

***Note:*** Since the sequencing alignments are derived from the HiFi Revio system, we select the “hifi_revio” model.

> mkdir models/hifi_revio

> wget -c -P models/hifi_revio/ -r -np -nH \

--cut-dirs=3 -A .pt \

https://www.bio8.cs.hku.hk/clair3/clair3_models_pytorch/hifi_revio/

***Note:*** Clair3 provides multiple pretrained models for various sequencing platforms and instruments. Users must select the appropriate model that matches their input sequencing reads. For other models, adjust the directory path and download URL accordingly. For example, if the sequencing data are generated from the ONT PromethION platform using R9.4.1 flow cells in Super Accuracy (SUP) mode, choose the “r941_prom_sup_g5014” model and modify the commands as follows:

> mkdir models/r941_prom_sup_g5014

> wget -c -P models/r941_prom_sup_g5014/ -r -np -nH \

--cut-dirs=3 -A .pt \

https://www.bio8.cs.hku.hk/clair3/clair3_models_pytorch/r941_prom_sup_g5014/

### Install software and set up environment

**Timing: 1–2 h**

6. Install the following tools: Clair3^12^ for SNV calling, LongPhase^13^ for read phasing, Samtools^14^ for alignment indexing, cuteHap^1^ for haplotype-resolved SV calling, and Truvari^15^ for benchmarking.

***Note:*** We recommend using conda or mamba for software installation, environment management, and package management.

> mamba create -n clair3_env -c conda-forge -c bioconda -y clair3

> mamba create -n longphase_env -c conda-forge -c bioconda \

-y longphase samtools

> mamba create -n cutehap_env -c conda-forge -c bioconda -y cutehap

> mamba create -n truvari_env -c conda-forge -c bioconda \

-y truvari samtools

## Key resources table

**Table:** 

REAGENT or RESOURCE	SOURCE	IDENTIFIER
**Deposited data**		

GRCh38 human reference genome	Genome Reference Consortium	GRCh38
HG002 sequencing read alignments	Pacific Biosciences	HG002-rep1 (Revio)

**Software and algorithms**		

Clair3 (v2.0.0)	Zheng et al.^12^	https://github.com/HKU-BAL/Clair3
miniSNV (v1.0)	Cui et al.^16^	https://github.com/CuiMiao-HIT/miniSNV
LongPhase (v2.0.1)	Lin et al.^13^	https://github.com/twolinin/longphase
WhatsHap (v2.8)	Martin et al.^17^	https://github.com/whatshap/whatshap
Bcftools (v1.23.1)	Danecek et al.^14^	https://github.com/samtools/bcftools
Samtools (v1.3.1)	Danecek et al.^14^	https://github.com/samtools/samtools
cuteHap (v1.0.4)	Cao et al.^1^	https://github.com/Meltpinkg/cuteHap
Truvari (v5.4.0)	English et al.^15^	https://github.com/ACEnglish/truvari

**Other**		

Linux server	N/A	N/A

## Materials and equipment

• Reference genome (FASTA file) of the human genome and long-read sequencing alignments (BAM file) of the sample.
• Required software for this protocol (including, at a minimum, Clair3, LongPhase, Samtools, cuteHap).

## Step-by-step method details

### Call SNVs in the sample

**Timing: 3–6 h**

Here, we describe steps for SNV calling, which requires the sequencing BAM file which contains the long-read alignments to the reference genome and the reference genome FASTA file as input, and outputs a VCF file listing all detected SNVs.

***Note:*** SNVs, which change a single nucleotide base (e.g., A→G), are the most abundant form of genomic variation. Because they are common and widely distributed across the genome, most read phasing tools rely on accurately detecting SNVs in a sample. These SNVs then serve as markers to distinguish which sequencing reads come from the paternal haplotype versus the maternal haplotype. In this protocol, we recommend using Clair3 to achieve accurate SNV detection. Clair3 is a well-tested caller for long-read data, but if users prefer another SNV caller (such as miniSNV,^16^ DeepVariant^18^ or Longshot^19^), that is also acceptable. For more detailed information about Clair3, including installation, usage examples, and parameter tuning, please refer to its GitHub repository.

1. Execute Clair3 within the corresponding Conda environment. Troubleshooting 2.

> conda activate clair3_env

> run_clair3.sh \

--bam_fn=alns/HG002.m84011_220902_175841_s1.GRCh38.bam \

--ref_fn=ref/GRCh38_full_analysis_set_plus_decoy_hla.fa \

--threads=16 --platform=hifi \

--model_path=models/hifi_revio --output=snvs

***Note:*** The “--platform” and “--model_path” parameters should be adjusted according to the platform and sequencer used to generate the sequencing alignments. Clair3 supports two long-read sequencing platforms: “hifi” and “ont”. It also provides multiple models, including “hifi_revio”, “hifi_sequel2”, “r941_prom_hac_g360+g422”, “r941_prom_sup_g5014”, and so on. These models are downloaded together with Clair3 into the directory “${CONDA_PREFIX}/bin/models/”. If the models are not found, they can be downloaded via https://www.bio8.cs.hku.hk/clair3/clair3_models_pytorch/ (as described in the download the dataset step). The choice of platform and model depends on the sequencing platforms and instruments of the input alignments.

***Alternatives:*** While we recommend Clair3 for SNV detection, users are free to use other SNV detectors, such as miniSNV, DeepVariant, Longshot, and so on. To help users get started with an alternative, we provide detailed instructions for installing and running miniSNV as a concrete example. Troubleshooting 3.

> mamba install -c conda-forge -c bioconda -y whatshap

> git clone https://github.com/CuiMiao-HIT/miniSNV.git

> cd miniSNV/Release

> make -j 12

> cd ../..

> mkdir minisnv_dir

> python miniSNV/chr_chunk_task.py \

--fin_ref ref/GRCh38_full_analysis_set_plus_decoy_hla.fa \

--fin_bam alns/HG002.m84011_220902_175841_s1.GRCh38.bam \

--workDir minisnv_dir --human \

--dup_bed miniSNV/GRCh38_segdups.bed \

--homo_bed miniSNV/GRCh38_AllHomopolymers_gt6bp_imperfectgt10bp_slop5.bed

### Phase read alignments using the detected SNVs

**Timing: 10–30 min**

Here, we describe steps for SNV calling, which requires the sequencing BAM file which is the same alignment file used for SNV calling, the reference genome FASTA file and the SNV callset VCF file as input, and outputs a tagged BAM file where each read now includes a haplotag.

***Note:*** The read phasing procedure takes the aligned sequencing reads and assigns a haplotag to each read. A haplotag is simply a label that indicates which haplotype the read originally came from – for example, whether it belongs to the paternal copy (inherited from the father), the maternal copy (inherited from the mother), or is ambiguous (labeled as unknown). In this protocol, we recommend using LongPhase to perform read phasing. LongPhase is specifically designed for long-read data and can efficiently handle the SNV markers identified in the previous step. However, if users prefer other long-read phasing tools (such as WhatsHap^17^), those are also acceptable. For more detailed information about LongPhase, including installation, usage options, please refer to its GitHub repository.

2. Execute LongPhase in phase mode to phase the SNV callset generated by Clair3.

> conda activate longphase_env

> longphase phase -s snvs/merge_output.vcf.gz \

-b alns/HG002.m84011_220902_175841_s1.GRCh38.bam \

-r ref/GRCh38_full_analysis_set_plus_decoy_hla.fa -t 16 \

-o phased/longphase --pb

***Note:*** The “--pb” parameter is used for PacBio sequencing alignments. If the input alignments are from ONT sequencing, change the parameter to “--ont”.

3. Execute LongPhase in haplotag mode to haplotag the input sequencing alignments and generate the tagged BAM file.

> longphase haplotag \

-r ref/GRCh38_full_analysis_set_plus_decoy_hla.fa \

-s phased/longphase.vcf \

-b alns/HG002.m84011_220902_175841_s1.GRCh38.bam -t 16 \

-o phased/longphase.tag

4. Execute Samtools to create an index for the tagged BAM file.

> samtools index phased/longphase.tag.bam

***Alternatives:*** While we recommend LongPhase for the read phasing step, the protocol is also compatible with other long-read phasing tools, such as WhatsHap, and so on. As an example, we provide detailed installation and execution instructions for WhatsHap here.

> mamba install -c conda-forge -c bioconda -y whatshap

> whatshap phase snvs/merge_output.vcf.gz \

alns/HG002.m84011_220902_175841_s1.GRCh38.bam \

--reference=ref/GRCh38_full_analysis_set_plus_decoy_hla.fa \

--distrust-genotypes --ignore-read-groups \

-o phased/whatshap.vcf.gz

> bcftools index phased/whatshap.vcf.gz

> whatshap haplotag phased/whatshap.vcf.gz \

alns/HG002.m84011_220902_175841_s1.GRCh38.bam \

--reference=ref/GRCh38_full_analysis_set_plus_decoy_hla.fa \

--ignore-read-groups -o phased/whatshap.tag.bam \

--output-haplotag-list=phased/phasedinfo.tsv

> samtools index phased/whatshap.tag.bam

### Call haplotype-resolved SVs using the tagged alignments

**Timing: 3–10 min**

Here, we describe steps for SV calling, which requires the tagged BAM file and the reference genome FASTA file as input, and outputs a VCF file containing all detected haplotype-resolved SVs.

***Note:*** The SV calling procedure detects SVs from the tagged BAM file that was produced by the read phasing step. SVs are large genomic alterations, typically spanning 50 base pairs or more. The protocol identifies five common types of SVs, including deletion (DEL), insertion (INS), inversion (INV), duplication (DUP), translocation (TRA), from the tagged BAM file and assigns phased genotypes to each detected SV. Because we use a haplotype-tagged BAM file, the output will be haplotype-resolved. Herein, we recommend using cuteHap for accurate SV detection. This tool is specifically designed to work with phased long-read data and can call all five SV types with high performance. For more detailed information about cuteHap, including installation, usage options, please refer to its GitHub repository.

5. Execute cuteHap to detect haplotype-resolved SVs from the tagged BAM file. Troubleshootings 4 and 5.

> conda activate cutehap_env

> cuteHap phased/longphase.tag.bam \

ref/GRCh38_full_analysis_set_plus_decoy_hla.fa \

svs/cutehap.vcf svs -s 5

***Note:*** The “-s” parameter specifies the minimum number of supporting reads required for an SV to be reported. In other words, if a candidate SV is seen in fewer reads than this threshold, cuteHap will ignore it to filter out false positives caused by sequencing errors or alignment artifacts. The optimal value for “-s” depends on the sequencing coverage and is recommended to be set to approximately one-sixth of the average coverage. cuteHap offers several other commands-line parameters that control various aspects of SV detection, such as minimum SV length, maximum distance between breakpoints, and filtering by read mapping quality. We have compiled a table of the parameters that are most likely to be used when running cuteHap (Table 1).

**Table 1:** A brief description, default value of each parameter in cuteHap

Parameter	Description	Default value
threads	Number of threads to use.	16
min_mapq	Minimum mapping quality value of alignment considered.	20
min_read_len	Minimum alignments of reads.	500
merge_del_threshold	Maximum distance of deletion signals to be merged.	0
merge_ins_threshold	Maximum distance of insertion signals to be merged.	0
min_support	Minimum number of reads that support an SV.	10
min_size	Minimum size of SV.	50
max_size	Maximum size of SV.	100000
mosaic	Enable to detect mosaic SVs.	False
max_cluster_bias_INS	Maximum distance to cluster reads together for INS.	1000
diff_ratio_merging_INS	Minimum basepair identity to merge breakpoints for INS.	0.9
max_cluster_bias_DEL	Maximum distance to cluster reads together for DEL.	1000
diff_ratio_merging_DEL	Minimum basepair identity to merge breakpoints for DEL.	0.9

## Expected outcomes

Throughout this document, we have presented a complete, step-by-step guide for accurately performing haplotype-resolved SV calling using cuteHap. The protocol is designed to overcome a common challenge: standard SV calling methods often cannot distinguish between the two copies of each chromosome. By leveraging long-read phasing technology, we improve upon traditional SV calling in accuracy and haplotype resolution. Phasing information reduces false positives and helps correctly assign variants to the correct haplotype, which enables users to obtain separate SV calls for each parental chromosome copy.

Importantly, this pipeline avoids the high time and computational costs associated with whole-genome assembly. Instead, we work directly from aligned reads, which is much faster and more resource-efficient. The entire protocol, from SNV calling through read phasing to final SV calling, completes within one natural day on typical datasets. Moreover, the pipeline is compatible with most modern CPU-based computing systems; users do not need hardware such as GPUs or large clusters. A standard multi-core server or even a powerful desktop computer with sufficient RAM (e.g., 32–64 GB) is usually adequate.

The pipeline processes long-read sequencing alignments and uses haplotype phasing information to enhance both SV detection and genotyping. The final output is a haplotype-resolved SV callset, a list of SVs with clear assignments to the two haplotypes. The protocol contains three main steps:

• Step 1 performs SNV calling to identify SNVs that serve as haplotype-specific markers. These markers will later guide the read phasing process. Clair3 is recommended in this step, although other long-read-based SNV callers such as miniSNV, DeepVariant, and Longshot are also acceptable. The expected output of this step is a directory containing the SNV calling results from Clair3, including a compressed VCF file merge_output.vcf.gz and its index file merge_output.vcf.gz.tbi.
• Step 2 performs read phasing to determine the haplotype origin of each alignment. This is done by assigning a haplotag to each read. LongPhase is recommended in this step, though other long-read-based phasing tools such as WhatsHap are also acceptable. The expected output is a haplotagged alignment file named longphase.tag.bam, where each read record includes an HP field, along with its index file longphase.tag.bam.bai.
• Step 3 performs haplotype-resolved SV calling of various types and assign genotypes using the haplotagged reads from Step 2. cuteHap is used in this step, and the expected output is a VCF file containing the sample’s haplotype-resolved SVs, along with the SV genotypes indicating which haplotype carries it. This file is the final result of the entire protocol.

## Quantification and statistical analysis

**Timing: 30–60 min**

After completing the above three-step protocol and obtaining a haplotype-resolved SV callset in VCF format, users can apply SV benchmarking to assess the quality of the detected SV callsets. The benchmark tool compares the SV calls against a trusted reference called the ground truth and produces standard performance metrics. Herein, the ground truth is a manually validated, high-confidence SV callset from HG002 T2T assembly. Along with the callset, the benchmark also uses corresponding high-confidence regions, which are the specific genomic intervals where the ground truth is considered reliable. We recommend using the widely-used SV benchmark tool, Truvari, to heuristically compare the detected SV callsets against the high-confidence ground-truth callset. From this comparison, Truvari calculates several performance metrics, including precision, recall, F1 score, and genotype concordance. These metrics quantitatively describe the quality of the detected SV callsets.

When evaluating the SV callset with Truvari, there are two main commands: an essential command “truvari bench”, which performs the direct comparison between the detected SV callset and the ground-truth callset, and an optional command “truvari refine”, which takes the output from “truvari bench” and refines the benchmark results by adjusting for differences in how the same structural variant might be represented. The refine step is not necessary for a simple evaluation, but it is recommended for achieving a more accurate and fair comparison, especially when comparing different SV callers that use different representation conventions. Troubleshooting 6.

> conda activate truvari_env

> bgzip -c svs/cutehap.vcf > svs/cutehap.vcf.gz

> tabix -p vcf svs/cutehap.vcf.gz

> truvari bench -b benchmarks/GRCh38_HG2-T2TQ100-V1.1_stvar.vcf.gz \

-c svs/cutehap.vcf.gz --includebed \

benchmarks/GRCh38_HG2-T2TQ100-V1.1_stvar.benchmark.bed \

-o truvari_cmp --pick ac --passonly -r 2000 -C 5000 \

-f ref/GRCh38_full_analysis_set_plus_decoy_hla.fa

> truvari refine --use-original-vcfs --align mafft \

-f ref/GRCh38_full_analysis_set_plus_decoy_hla.fa \

-r truvari_cmp/candidate.refine.bed truvari_cmp -t 16 \

--mafft-params “--auto --thread 16”

***Note:*** The “-b” and “--include_bed” parameters in the “truvari bench” command specify the ground truth for the sample being evaluated. Both the ground truth VCF and the high-confidence BED file must be consistent with the sample evaluated and the reference genome used throughout the pipeline. The inconsistence will make the metrics meaningless. In this protocol, we use the HG002 sample and the GRCh38 reference genome as example.

**Figure 1:**
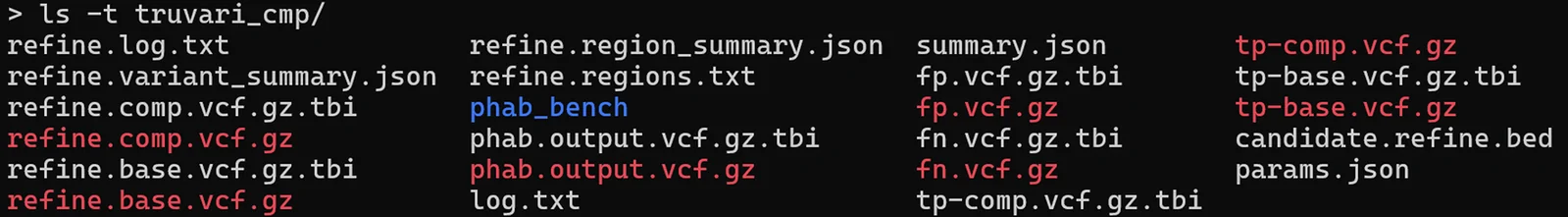
The directory structure of the output directory of Truvari

**Figure 2:**
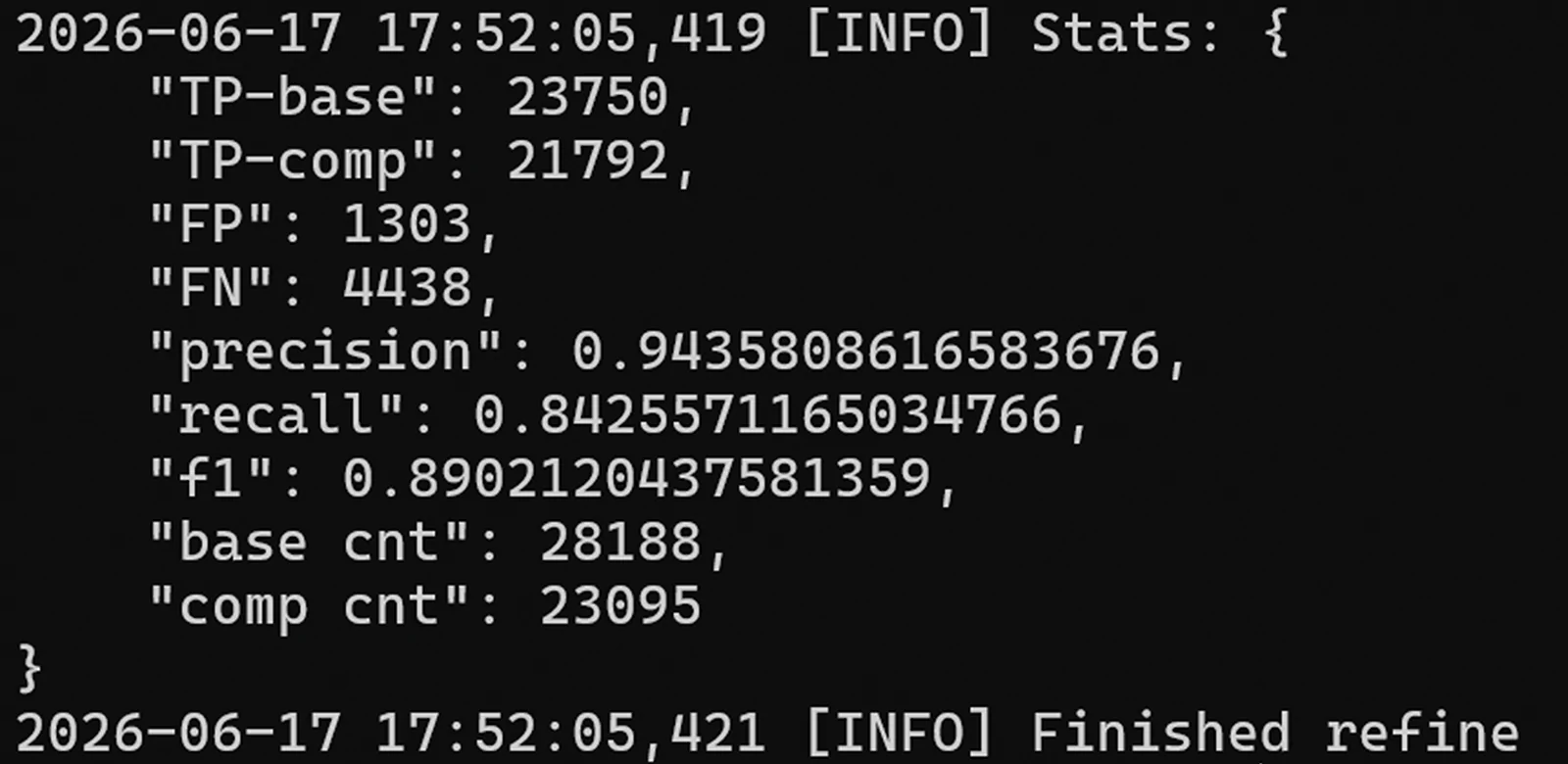
The evaluation results of Truvari, which include the precision, recall, F1

The output directory named “truvari_cmp” contains several benchmark files that allow users to inspect the results at the individual SV level. The layout of this directory is shown in Figure 1. The output directory contains the following compressed VCF files to enable the concrete observation of benchmark, including the true positives in the detected callsets, true positives in the ground truth, false positives, and false negatives. In addition to these compressed VCF files, it also includes two JSON metric files that summarize the performance. The first is the “summary.json” file, which includes the results of “truvari bench”. It represents a direct comparison between the detected SV callsets and the ground truth. The metrics here include precision, recall, F1 score, genotype concordance, and counts of true positives, false positives, and false negatives. The second is the “refine.variant_summary.json” file, which contains the results after refinement, where Truvari has adjusted for consistency in variant representation Figure 2. The metrics here include precision, recall, F1 score.

## Limitations

This protocol is designed to be user-friendly and runs entirely via command-line commands on Linux systems. It enables fast and accurate haplotype-resolved SV detection. If we exclude the one-time steps of dataset download and environment setup, the complete pipeline typically finishes in just a few hours.

However, it is important to note that the overall performance of this protocol depends on the choice of bioinformatics tools. For demonstration, we selected three advanced tools—Clair3, LongPhase, and cuteHap. However, other state-of-the-art tools with similar functions can be used as direct replacements, and they are expected to yield results with no significant differences. In other words, the protocol is flexible and does not rely on a single specific set of software.

In addition, though the protocol uses a human sample as its example, it is equally suitable for any other diploid sample (e.g., from other animals or plants with two sets of chromosomes). However, samples with polyploid genomes are not applicable. The reason is that polyploidy interferes with the phasing procedure, which is designed to separate reads into exactly two haplotypes (maternal and paternal). With three or more haplotypes, accurate phasing of current phasing tools becomes impossible.

Finally, although short-read-based phasing tools do exist, their performance in haplotype-resolved SV detection is limited. Short reads cannot phase across long, complex structural variants reliably. Therefore, this protocol is designed to work only with long-read sequencing alignments.

## Troubleshooting

### Problem 1

Due to potential issues in the download process such as an unstable internet connection, the server sending a partial file, or other errors, input files for the protocol may be corrupted and incomplete (related to the download the dataset section).

### Potential solution

To avoid issues with corrupted or incomplete input files, use the “-c” option when downloading datasets. This option tells the downloader to check if a partial file already exists on the disk; if so, it will continue downloading from the point of interruption rather than starting over from the beginning. Also, after the download is complete, check the MD5 checksum of the downloaded file with the one provided by the source (if provided), to ensure the correctness of the file.

### Problem 2

Clair3 exits immediately after the environment checking and reports following WARNING: “No contig intersection found, output header only in snvs/merge_output.vcf.gz” (relate to the call SNVs in the sample section).

### Potential solution

This problem occurs when the input reference genome does not match the reference genome that was used to generate the alignment file. In other words, the contig names or sequences are inconsistent between the two. To resolve this, make sure the downloaded reference genome version is exactly the same as the one to which the reads were originally mapped. If users are unsure which reference genome was used for alignment, check the BAM file header using a tool like “samtools view -H″ where typically records the reference contig names and the genome information. Matching the reference files is essential for Clair3 to find overlapping contigs and produce proper SNV calls.

### Problem 3

When compiling miniSNV, users may see the following error message: “./src/cmtools/form.hpp:35:5: error: ‘uint32_t’ does not name a type” (relate to the call SNVs in the sample section).

### Potential solution

This error occurs because miniSNV used in the protocol was originally developed with GCC version 4.8.5. If the system uses a newer GCC version, the compiler may be stricter and cause the uint32_t type not to be recognized. To fix this, manually add the missing header. Open the file “miniSNV/src/cmtools/form.hpp” using any text editor (such as vim, nano), then insert the following line at the top of the file (together with other #include lines): “#include <cstdint>”. After saving the file, recompile miniSNV through “make -j 12”. The error should be resolved.

### Problem 4

When running cuteHap, users may encounter a KeyError similar to: “KeyError: ‘GL000008.2'” (relate to the call haplotype-resolved SVs using the tagged alignments section).

### Potential solution

This error indicates that the input reference genome does not contain a contig named GL000008.2, but the aligned reads include alignments to that contig. In other words, the reference provided to cuteHap is not fully concordant with the reference used to generate the BAM file. To resolve this, ensure that the reference genome FASTA file provided to cuteHap is exactly the same version and includes all contigs that appear in the BAM header. If a particular contig is still missing, either download a complete reference or re-align the reads using a consistent, full reference.

### Problem 5

When running cuteHap, users may see a FileNotFoundError like: “FileNotFoundError: [Errno 2] No such file or directory: ‘xxx.pickle'” (relate to the call haplotype-resolved SVs using the tagged alignments section).

### Potential solution

This error is typically caused by race conditions when cuteHap uses multiple parallel threads. To resolve this, simply reduce the number of threads and re-run cuteHap. This minimizes file-access conflicts and usually eliminates the error without affecting final results.

### Problem 6

In the optional “quantification and statistical analysis” section, when running Truvari refine, users may encounter the error like:

“[E::fai_build_core] File truncated at line 1

pysam.utils.SamtoolsError: ‘samtools returned with error 1: stdout=, stderr=[faidx] Could not build fai index/tmp/xxxx.fa.fai'” (relate to the quantification and statistical analysis section).

### Potential solution

This error arises when truvari refine invokes pysam.samtools.faidx to extract reference subsequences. In pysam versions ≥ 0.20, the FASTA output is directed to stdout instead of a file, causing large FASTA chunks to be printed directly to the terminal and leading to failure. While downgrading pysam could be a workaround, Truvari has released a develop version that fixes this error without altering other dependencies. We therefore recommend installing this develop version using the commands below to resolve this potential problem.

> mamba create -n truvari_develop -c conda-forge -c bioconda \

-y pyabpoa mafft

> git clone https://github.com/ACEnglish/truvari.git

> cd truvari

> python3 -m pip install .

## Resource availability

### Lead contact

Further information and requests for resources and reagents should be directed to and will be fulfilled by the lead contact, Tao Jiang (tjiang@hit.edu.cn).

### Technical contact

Technical questions on executing this protocol should be directed to and will be answered by the technical contact, Shuqi Cao (sqcao@stu.hit.edu.cn).

### Materials availability

No materials were generated in this study.

### Data and code availability

Source data analyzed in the paper are available in the PacBio cloud database (https://downloads.pacbcloud.com/public/revio/2022Q4/HG002-rep1/analysis/) and the 1000GP FTP website (http://ftp.1000genomes.ebi.ac.uk/vol1/ftp/technical/reference/GRCh38_reference_genome/). All tools used in the analysis are open source in GitHub. The accession number for the generated SV callsets reported in this paper is Zenodo: https://doi.org/10.5281/zenodo.19728430.

## Acknowledgments

This work has been supported by the 10.13039/100014718National Natural Science Foundation of China (no: 62472120), the 10.13039/501100012166National Key Research and Development Program of China (no: 2024YFC3406303), and the 10.13039/501100012226Fundamental Research Funds for the Central Universities (no: XNJKKGYDJ2026010). This work has also been partially supported by 10.13039/501100003472Harbin Institute of Technology Kunpeng&Ascend Center of Cultivation.

## Author contributions

S.C. and T.J. conceived and constructed the protocol. S.C. and C.W. prepared scripts in each step. C.W. and Y.H. implemented and validated the whole pipeline. S.C. and T.J. wrote the manuscript. The authors read and approved the final protocol.

## Declaration of interests

The authors declare no competing interests.
